# An Exceptional Case of Diplopia and Ptosis: Extramedullary Plasmacytoma of the Clivus With Multiple Myeloma

**DOI:** 10.7759/cureus.23219

**Published:** 2022-03-16

**Authors:** Syed Hamza Bin Waqar, Aiman Rehan, Navid Salahi, Li Zhonghua, Isabel McFarlane

**Affiliations:** 1 Internal Medicine, State University of New York Downstate Health Sciences University, New York, USA; 2 Internal Medicine, Dow University of Health Sciences, Karachi, PAK; 3 Pathology, State University of New York Downstate Health Sciences University, New York, USA; 4 Pathology, Kings County Hospital Center, New York, USA

**Keywords:** extramedullary plasmacytoma, intracranial mass, clivus, diplopia, multiple myeloma

## Abstract

Intracranial plasmacytoma is an exceedingly rare presentation of plasma cell neoplasms. Usually presenting late in the course of the disease, progression from the presentation can be abrupt. Hence, a low threshold to biopsy the lesion should be maintained during diagnostic evaluation. Multiple myeloma workup should also be sent and treated concomitantly along with local treatment. Here, we present a case of extramedullary plasmacytoma of the clivus leading to progressive visual deficits with undiagnosed multiple myeloma requiring pulse steroids, intracranial irradiation, and high-dose chemotherapy with improvement in symptoms.

## Introduction

Plasmacytomas involving the clivus are an extremely rare phenomenon. They arise from a localized proliferation of plasma cells in the bone or soft tissue. According to the World Health Organization, plasmacytomas can be categorized into solitary bone plasmacytomas and extramedullary plasmacytomas [1]. The two types can be distinguished based on the sites of origin, conversion to multiple myeloma, and prognosis. Intracranial plasmacytoma involving the skull base has been reported only a few times in the literature [2]. Here, we report the case of a 60-year-old man who presented with diplopia and ptosis with progressive worsening of visual field acuity and was found to have a clival mass which was later diagnosed as plasmacytoma. Further management revealed an underlying immunoglobulin (Ig)G lambda multiple myeloma with extramedullary plasmacytoma. Symptoms resolved with steroid pulses and radiation to the clivus.

## Case presentation

A 60-year-old African American man with a medical history of diabetes mellitus and hypertension presented to our tertiary care hospital with visual changes, including blurriness and double vision with a drooping eyelid of the left eye. The patient had no other complaints, including no floaters or flashes. A detailed visual assessment showed ptosis of the left eye with anisocoria, exotropia, and horizontal nystagmus to the right gaze. Computed tomography (CT) of the brain was performed, which revealed a 3.3 cm × 3.2 cm × 3.5 cm enhancing suprasellar mass with associated osseous erosion and extension to the left cavernous sinus. Possible differentials of chordoma, lymphoma, metastatic disease, or plasmacytoma were made. Magnetic resonance imaging (MRI) of the brain and orbit with and without contrast was performed to characterize the mass better and reveal the relationship to orbit content.

**Figure 1:**
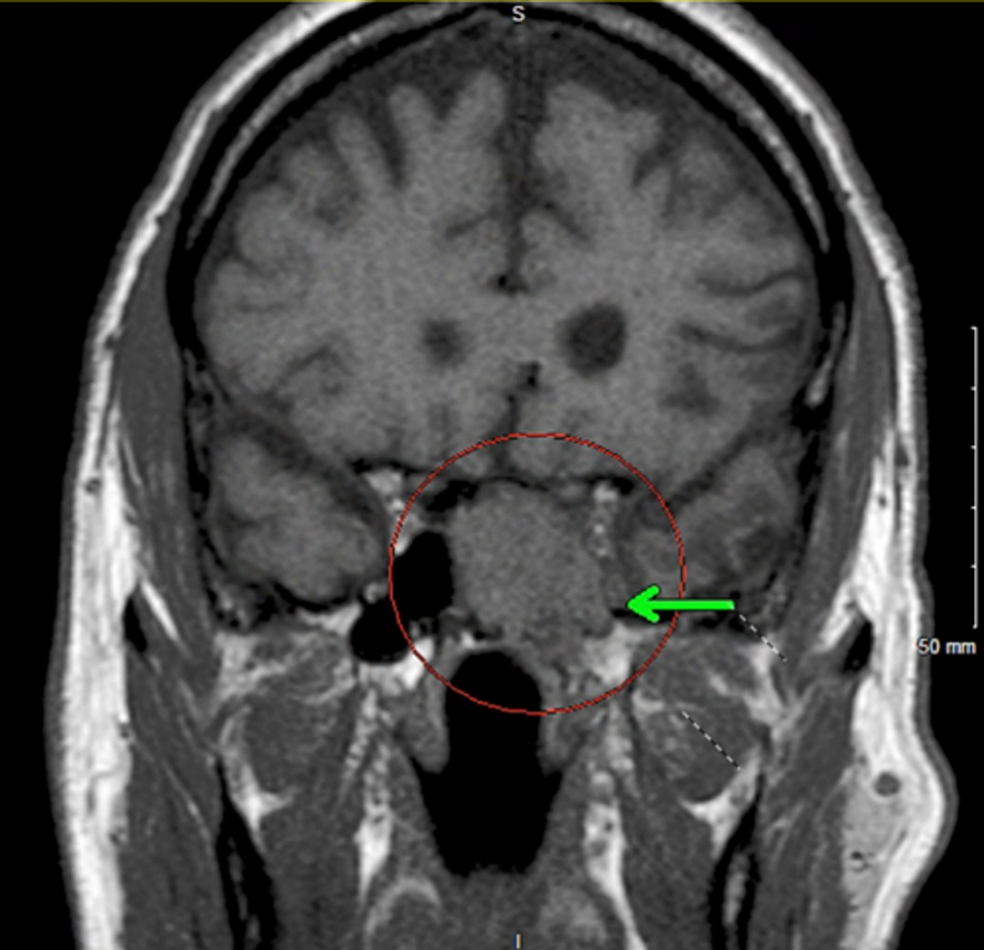
Coronal T1-weighted MRI sequence showing a mass in close proximity to the inferomedial aspect of the left optic canal (green arrow). MRI: magnetic resonance imaging

**Figure 2:**
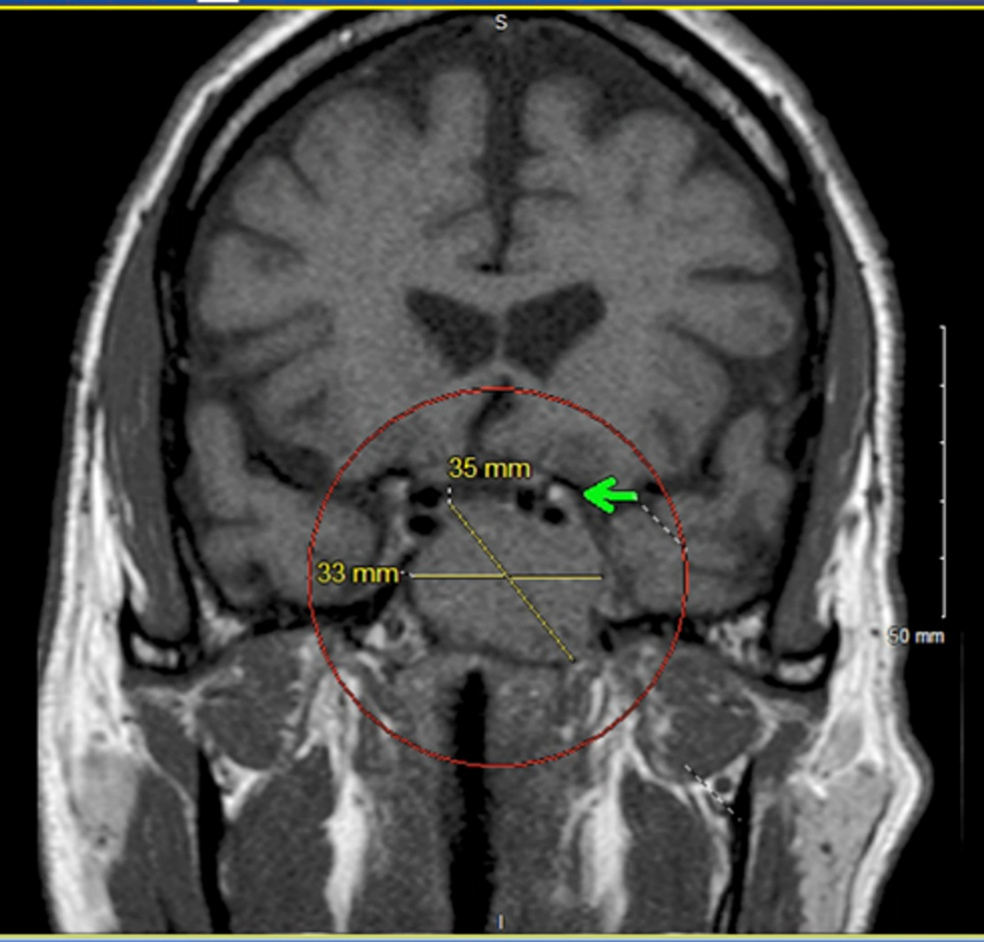
Coronal T1-weighted MRI sequence showing hypointense 35 mm × 33 mm clivus mass extending to partially encase cavernous segment of the internal carotid artery (green arrow). MRI: magnetic resonance imaging

MRI revealed a large infiltrative destructive lesion centered within the clivus with hypointensity and mild hyperintensity with avid enhancement on postcontrast sequences on T1 and T2 series, respectively. The mass was 35 mm × 33 mm × 35 mm with the destruction of a large portion of the clivus, with greater involvement of the left side where tumor infiltration extended further posteriorly. Invasion of the left cavernous sinus with partial encasement of the cavernous segment of the left internal carotid artery was seen with the tumor widening the left carotid siphon (Figures 1, 2).

**Figure 3:**
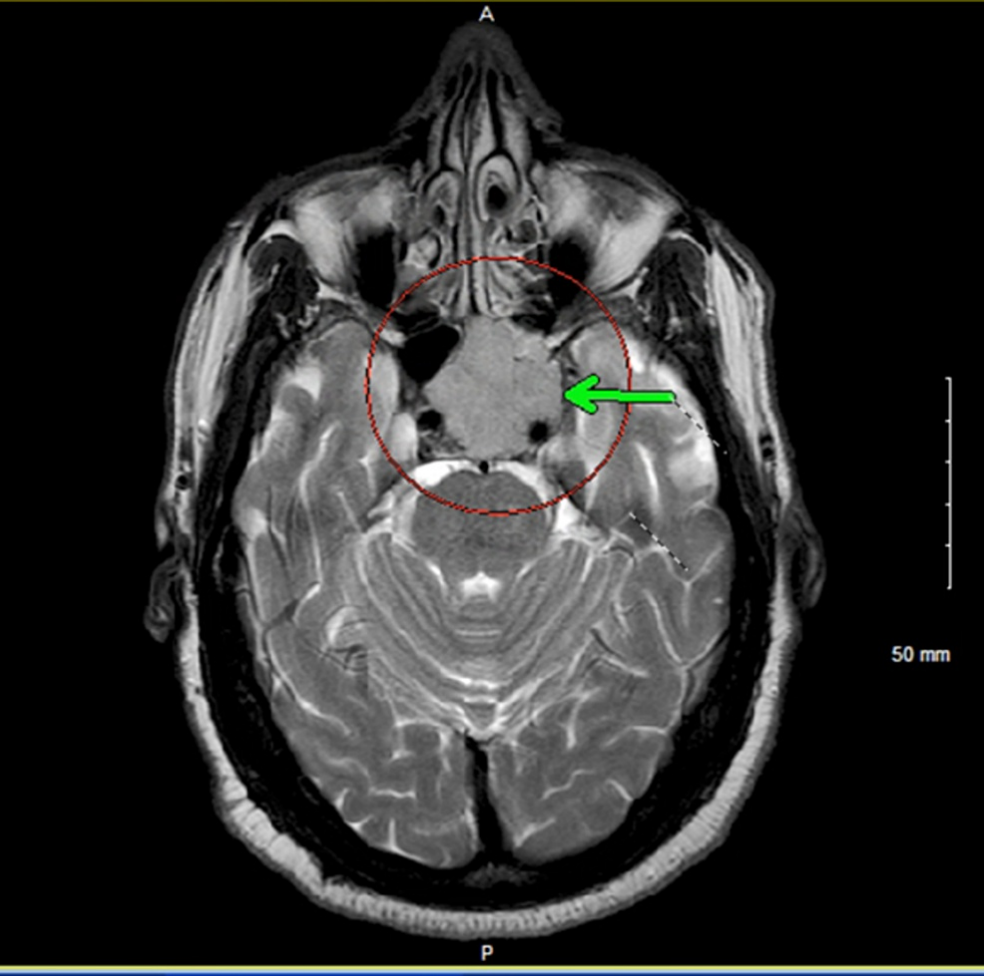
Axial T2-weighted MRI sequence showing the relationship of the mass with the optic nerve (green arrow). MRI: magnetic resonance imaging

The tumor was closely approximating the inferomedial margin of the left optic canal with no visible mass effect on the optic nerve or optic chiasm. Intra and extraconal spaces along with the intraorbital optic nerve were normal (Figure 3).

Findings were suggestive of plasmacytoma over other differentials. On day three of the presentation, the patient had worsening visual deficits. There was progression to fixed and dilated pupil, which was previously reactive with limitation of extraocular motion in all dimensions of the left eye and more pronounced adduction deficit. Humphrey visual field test showed a severe bilateral reduction in both eyes and a superior altitudinal deficit in the left eye.

**Figure 4:**
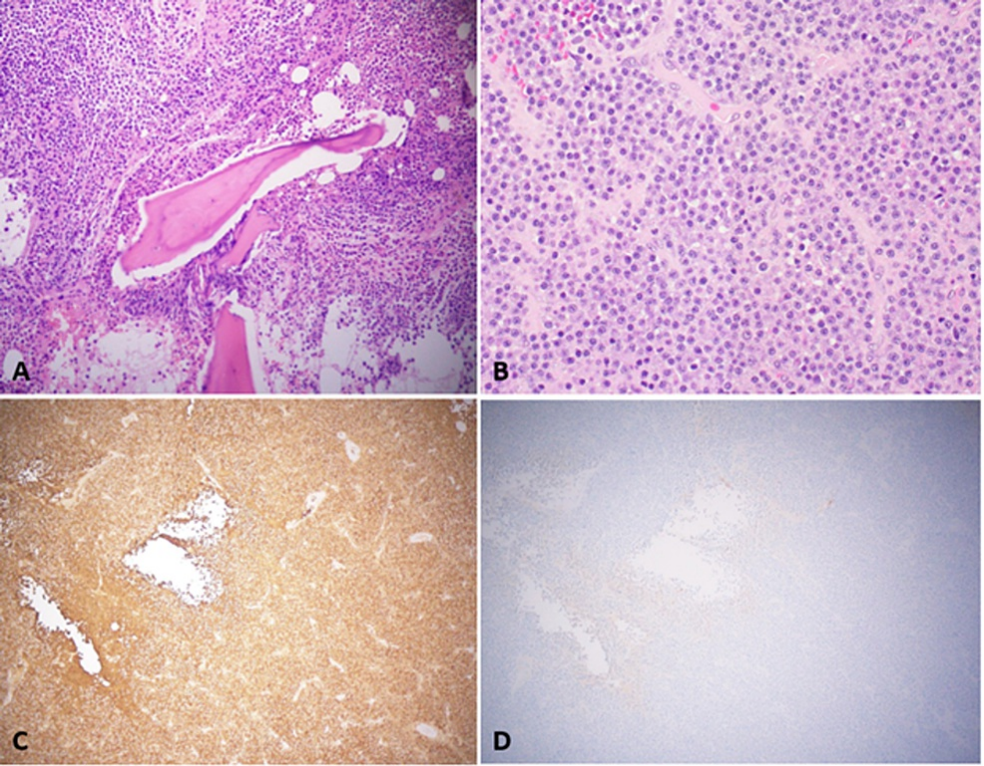
H&E stain shows sheets of plasma cells in the section from bone marrow (A: H&E, 200×) and the section from the skull base mass (B: H&E, 400×), with lambda light chain restriction. The plasma cells are positive for lambda (C: 100×) but negative for kappa (D: 100×) by immunohistochemistry. H&E: hematoxylin and eosin

Given the progression of visual field defects, a decision was made to arrange emergent transsphenoidal biopsy, which revealed sheets of plasma cells with lambda light-chain restriction consistent with plasmacytoma. Bone marrow biopsy was performed again to diagnose multiple myeloma, which revealed sheets of plasma cells with lambda restriction without staining for kappa light chain on immunohistochemistry. Patchy moderate to a marked increase in reticulin fibers were seen on reticulin stain with an increase in stainable iron on the iron stain (Figures 4A-4D).

.Multiple myeloma workup was concomitantly sent, and the findings are listed in Table 1.

**Table 1:** Multiple myeloma laboratory findings. IFE: immunofixation electrophoresis; FLC: free light chain; Ig: immunoglobulin

Laboratory	Patient’s value	Normal range
Calcium	9.5 mg/dL	8.2–10.0 mg/dL
Serum creatinine	0.84 mg/dL	0.7–1.3 mg/dL
Lactate dehydrogenase	350U/L	150–300 U/L
Hemoglobin	13.1 mg/dL	14–16 mg/dL
Hematocrit	41%	42–50%
Total Protein	11.7 mg/dL	6.0–8.3 mg/dL
Albumin	2.2 mg/dL	3.50–5.70 mg/dL
Alkaline phosphatase	169 U/L	34–104 U/L
Beta-2-microglobulin	2.2 µg/mL	0.80–2.20 µg/mL
Urine protein electrophoresis M-spike	11.1%	Negative
24-hour urine protein	290 mg	<100 mg
Estimated Bence-Jones protein in urine	32 mg	Negative
Immunofixation electrophoresis/FLC assay		
Serum protein electrophoresis M-spike	3.8 g	Negative
IgG	6,038 mg/dL	610–1,680 mg/dL
IgA	149 mg/dL	84–499 mg/dL
IgM	108 mg/dL	35–242 mg/dL
Ig kappa FLC	1.16 mg/dL	0.33–1.94 mg/dL
Ig lambda FLC	11.81 mg/dL	0.57–2.63 mg/dL
Kappa/Lambda FLC ratio	0.10	0.26–1.65

Pan-CT was performed and revealed multiple lytic lesions in the bilateral femur and vertebral bodies. Hence, the diagnosis of IgG lambda multiple myeloma with extramedullary plasmacytoma was made. Cytogenetics revealed a translocation between chromosomes 4 and 14 (4;14).

The patient received a total of 160 mg pulse dexamethasone over four days with a prolonged taper. Radiation was completed for a total of 3,500 cGy to the base of the skull over 28 days. Treatment with lenalidomide, bortezomib, and dexamethasone was planned. The patient had significant improvement in his visual field deficits with the resolution of anisocoria and ptosis.

## Discussion

Plasmacytomas are malignant tumors arising from the monoclonal proliferation of plasmacytes. They often show secretion of IgA and IgG and can be closely associated with underlying multiple myeloma [3]. Solitary intracranial plasmacytomas (SIPs) are extremely infrequent. Their mean age of presentation is 57 years, with others ranging from 18 to 82 years [4]. Our case was also close to the mean age. Comparing the occurrence in both genders, SIPs occur in both males and females equally. However, extracranial solitary plasmacytomas have been observed more commonly in females [4,5]. Moreover, solitary plasmacytoma of the bone is seen in a 2:1 ratio, being more routinely found in males, as reported by Soutar et al. [5].

As seen in previous studies, the most common location for an SIP is the frontal calvaria [6,7]. Other preferential sites include the orbital tip, sphenoidal sinus, and the dorsum sellae. There have been very few reported cases of clival plasmacytomas in the literature. According to previous case reports, the mean age of occurrence in the case of a clival plasmacytoma is 57 years, with a stronger female predilection [8]. At the time of diagnosis, the presence of a concomitant peripheral extramedullary myeloma in bones such as the sternum or vertebrae has often been seen [5].

Often, SIP remains asymptomatic for a long time before any symptoms are seen. The first few presenting symptoms of an SIP include visual field defects, diplopia, bone invasion, and nausea and vomiting [4,9]. There can also be endocrinological problems owing to the sellar invasion. Involvement of the clivus can lead to diplopia because of compression of the sixth cranial nerve [10].

Radiology findings in previous studies have demonstrated that clival plasmacytomas show iso-intensity on both T1- and T2-weighted images, and a CT scan is likely to show signs of bone destruction. Initial differential diagnoses include chordoma, meningioma, pituitary adenoma, lymphoma, and metastases [11]. Positron emission tomography with fluorodeoxyglucose has also helped detect other medullary and extramedullary diseases [12]. Due to these nonspecific findings, histopathology has been used as the mainstay of diagnosis in plasmacytomas and was employed in diagnosing our patient.

In the case of a plasmacytoma, histological evaluation demonstrates the diffuse or sheet-like proliferation of plasma cells. This monoclonal division shows atypia, prominent nucleoli, a high nuclear to cytoplasmic ratio, and dispersed chromatin [13]. The nuclei are eccentrically placed with clock face chromatin and a clear area (halo). There is also positive staining for immunological kappa light chains. Immunohistochemical staining positive for CD138, 38, and 56 is often observed. Plasma cells can be explicitly identified by CD138, and malignant plasmacytes often stain positive, especially for CD56 in about 70% of cases [14].

Treating such a malignancy poses a challenge. SIP that involves the base of the skull can only be partially resected. Radiotherapy with routine follow-up can be employed to prevent its progression and has been proven to be successful because plasma cells are highly radiosensitive. Several studies have recommended treatment of SIP using 40-50 Gy fractionated radiotherapy. Strict oncologic surveillance is required in such patients to prevent the progression to multiple myeloma. If there is also co-existing multiple myeloma, the use of high-dose chemotherapy and its clinical management according to guidelines is required [15,16].

Transnasal endoscopic management has become reasonably popular over recent years. It has replaced the more invasive open surgical methods, especially in clivus or sphenoid sinus lesions. This allows for enhanced visualization and increased surgical confidence. A previous case series demonstrated the use of endoscopic techniques to effectively diagnose lesions in these areas, in which two were diagnosed as plasmacytomas of the clivus [17]. The role of surgery in such cases is now mostly restricted to obtaining a biopsy. In our case, a transsphenoidal biopsy was performed as well.

This case highlights the importance of considering plasmacytoma as a possible diagnosis when a lesion of the skull base is suspected and is found to be involving the cranial nerves. Although rare, plasmacytomas should be ruled out as a possible differential. The patient should also be evaluated for multiple myeloma and followed up routinely in case of its presence.

## Conclusions

This case illustrates an interesting presentation of plasmacytoma of the clivus with visual field deficits. It is prudent not to delay diagnosis as it can worsen deficits, and a minimally invasive approach should be opted for biopsy of the desired site. A dynamic approach with multidisciplinary team consultation should be utilized. Radiation and pulsed-dose steroids can be used once biopsy has been obtained, and treatment of the underlying multiple myeloma should be performed if diagnosed.
